# Dual Targeting of PI3K and HDAC by CUDC-907 Inhibits Pediatric Neuroblastoma Growth

**DOI:** 10.3390/cancers14041067

**Published:** 2022-02-20

**Authors:** Rameswari Chilamakuri, Saurabh Agarwal

**Affiliations:** Department of Pharmaceutical Sciences, College of Pharmacy and Health Sciences, St. John’s University, New York, NY 11439, USA; rameswari.chilamakuri19@my.stjohns.edu

**Keywords:** drug repurposing, PI3K pathway, epigenetics, Fimepinostat, neuroblastoma, pediatric cancer

## Abstract

**Simple Summary:**

High-risk neuroblastoma (NB) is an aggressive cancer of very young children and accounts for almost 15% of all pediatric cancer deaths. Current therapies include high-dose chemotherapy and radiation, which have long-term toxic side effects. Despite these intensive therapies, the overall 5-year survival rate of NB is less than 50%. Therefore, developing novel therapeutic approaches targeting the molecular mechanisms that drive NB progression is very important. In the present study, we repurpose CUDC-907, a dual inhibitor of PI3K and histone deacetylases. These regulators are known to regulate MYCN expression, a key prognostic marker of NB. CUDC-907 potently inhibits NB growth and 3D spheroid tumor growth by inhibiting PI3K, HDAC, and MYCN. Overall, our pre-clinical data demonstrate that repurposing CUDC-907 as a single drug is a novel and effective therapeutic approach for NB.

**Abstract:**

The dysregulation of PI3K, HDACs, and MYCN are well known for promoting multiple cancer types, including neuroblastoma (NB). Targeting the upstream regulators of MYCN, including HDACs and PI3K, was shown to suppress cancer growth. In the present study, we analyze different NB patient datasets to reveal that high *PI3K* and *HDAC* expression is correlated with overall poor NB patient survival. High *PI3K* level is also found to be associated with high *MYCN* level and NB stage progression. We repurpose a dual inhibitor CUDC-907 as a single agent to directly target both PI3K and HDAC in NB. We use in vitro methodologies to determine the efficacy and selectivity of CUDC-907 using six NB and three control fibroblast cell lines. Our results show that CUDC-907 significantly inhibits NB proliferation and colony growth, induces apoptosis, blocks cell cycle progression, inhibits MYCN, and enhances H3K9Ac levels by inhibiting the PI3K/AKT signaling pathway and HDAC function. Furthermore, CUDC-907 significantly inhibits NB tumor growth in a 3D spheroid tumor model that recapitulates the in vivo tumor growth. Overall, our findings highlight that the dual inhibition of PI3K and HDAC by CUDC-907 is an effective therapeutic strategy for NB and other MYC-dependent cancers.

## 1. Introduction

High-risk neuroblastoma (NB) is the most common extracranial solid tumor that accounts for almost 10% of all childhood-related cancers [1]. Despite major advancements in intensive multi-modal therapies, the overall 5-year survival rate of NB is less than 50% [2]. Current NB therapy follows an induction chemotherapy regimen that is often associated with disease comorbidities and increased risk of secondary malignancies [3]. Therefore, developing novel single-agent therapeutic strategies targeting multiple oncogenic pathways such as PI3K/AKT, histone deacetylases, and MYCN, is important for NB and other cancers.

The pathologic activation of *MYCN* plays a central role in NB, with *MYCN* amplification identified in approximately 25% of primary NB tumors [4]. *MYCN* amplification drives rapid metastasis, relapse, and drug resistance, and patients with relapsed *MYCN* amplified tumors have less than 5% overall survival rate [5]. Direct MYCN targeting strategies were shown to have limited effect, thus indirectly targeting the oncogenic activation of *MYCN* transcription is an important therapeutic approach for MYCN-driven cancers [4]. Among others, histone deacetylases (HDAC) and phosphoinositide-3 kinase (PI3K) are upstream regulators of *MYCN* and are also known to promote NB pathogenesis [6]. PI3K/AKT pathway is one of the most important intracellular signaling pathways regulating cell growth, proliferation, angiogenesis, motility, survival, and metabolism [7]. The PI3K signaling pathway also regulates multiple cellular proteins, such as mTORC1, S6 kinase, GSK3β, FOXO, MDM2, MYCN, P27, and BAD [8,9,10]. The dysregulation of PI3K has been reported in multiple cancer types, including prostate cancer, breast cancer, ovarian cancer, and NB [11,12].

Epigenetic alterations, such as histone acetylation and methylation, play an essential role in tumor initiation and progression [6]. HDACs are key transcription cofactors that regulate histone and non-histone protein substrates through epigenetic or non-epigenetic modifications, thus affecting multiple signaling networks, including MYC, p53, and STAT3 [13]. HDACs also play an important role in cell proliferation, differentiation, cellular homeostasis, and stemness maintenance [13]. Studies showed that the H3K9 acetylation (H3K9Ac) of active promoters positively correlates with gene expression, and HDAC inhibitors induce these acetylation marks by inhibiting HDAC enzymes [14]. HDAC inhibitors alone or in combination with other anti-cancer compounds showed promising preclinical results in the treatment of different cancers [15]. Preclinical studies have shown promising anti-cancer activity for combining PI3K inhibitors with HDAC inhibitors in various cancers [16]. 

In the present study, we use a dual PI3K and HDAC inhibitor, CUDC-907 or Fimepinostat, and show its potency in inhibiting NB growth. CUDC-907 is a first-in-class, oral small molecule dual HDAC (class I and II) and PI3K (class Iα, β, and δ) inhibitor that can simultaneously target multiple oncogenic signaling pathways [17,18]. CUDC-907 is effective in multiple cancer types, including acute myeloid leukemia, relapsed or refractory diffuse large B-cell lymphoma, prostate cancer, thyroid cancer, and multiple myeloma [19,20,21]. FDA designated CUDC-907 for fast track development for patients with relapsed diffuse large B-cell lymphoma [17]. Our preclinical results in NB demonstrate that CUDC-907 potently inhibits NB growth by inhibiting the PI3K signaling pathway and HDACs. We also found that CUDC-907 inhibits MYCN and enhances the H3K9 histone acetylation. Overall, our study highlights the efficacy of CUDC-907 as a single drug in targeting multiple pathways and as a novel therapeutic approach for NB. As CUDC-907 is currently under extensive clinical trials for multiple cancers, our study will further pave the way for the effective clinical translation of CUDC-907 for NB patients. 

## 2. Materials and Methods

### 2.1. Cell Culture and Reagents

Human NB cell lines NGP, LAN5, CHLA-255-MYCN (*MYCN*-amplified), SH-SY5Y, SK-N-AS, and CHLA-255 (*MYCN* non-amplified), and normal fibroblast control cell lines WI-38, NIH-3T3, and COS-7 were routinely cultured and maintained as described previously [22,23]. All cell lines used in this study were routinely tested for mycoplasma and validated via short-tandem repeat analysis for genotyping. In this study, the experiments were performed using different numbers of cell lines as required for the assay. Primary antibodies anti-PI3K (4292S), anti-p-PI3K (4228S), anti-PDK1 (3062S), anti-p-PDK1 (Ser241; 3438S), anti-AKT (9272S), anti-p-AKT (Thr308; 9275S), anti- p70S6 kinase (S6K; 9202S), anti-p-p70-S6 kinase (pS6K; Thr389; 9205S), anti-MYCN (9405S), anti-H3 (4499S), anti-H3K9Ac (9649S), anti-Cyclophilin B (43603S), and anti-rabbit IgG HRP-linked secondary antibody (7074S) were purchased from Cell Signaling Technology. CUDC-907 was purchased from MedChem Express, Monmouth Junction, NJ, USA.

### 2.2. Clinical Patient Dataset

NB patient datasets were analyzed using the publicly available patient databases from the R2: Genomic Analysis and Visualization Platform. Available online: https://hgserver1.amc.nl/cgi-bin/r2/main.cgi (accessed on 1 February 2022). This unique platform provides a multi-parametric analysis of NB patient outcomes with correlation to gene expression and microarray profiles of their primary tumors. In the present study, we analyzed a total of 1235 primary NB patient data by analyzing Versteeg, Kocak, and SEQC datasets. Dataset analyses were performed by including patients’ confounding factors.

### 2.3. Cell Viability and Clonogenic Assay

Cell viability assays were performed as described previously [23]. Briefly, cells were treated with different drug concentrations for 72 h, followed by incubation with CellTiter 96 AQueous One Solution from the Cell Proliferation Assay kit (G3582; Promega, Madison, WI, USA) as per the manufacturer’s instructions. Cell viability was measured using a spectrophotometer (SpectraMax iD3, Molecular Device, San Jose, CA, USA) at 490 nm. The data were analyzed using GraphPad Prism 9 software, and IC_50_ values were calculated for individual cell lines. Clonogenic cell colony formation assays were performed as described previously [23,24].

### 2.4. Apoptosis and Cell Cycle Assay

Apoptosis assays were performed using the Muse Annexin V and Dead Cell Kit (MCH100105; Luminex Corp., Austin, TX, USA), and cell cycle analysis was performed using Muse Cell Cycle Kit reagent (MCH100106; Luminex Corp), according to the manufacturer’s instructions and as described previously [23]. NB cell lines were treated for 16 h with different concentrations of CUDC-907. Apoptosis and cell cycle samples were analyzed using the Guava Muse cell analyzer (Luminex Corp) to determine the percentage of early apoptosis and cell cycle changes, respectively.

### 2.5. 3D Spheroid Tumor Assay

Three-dimensional spheroidal tumor assay was performed using three-dimensional spheroidal 96-well microplates (4515; Corning Inc., Glendale, AZ, USA), according to the manufacturer’s instructions and as described previously [23]. Briefly, NB spheroids of about 250 μm were formed, randomized, and treated with regular drug replenishment. Spheroid images were captured, and size was measured using a Leica DMi1 microscope using the LASX software suite from Leica Microsystems. This software suite provides tools to analyze the captured images and measure size. Each treatment cohort includes at least 6 spheroid tumors. A Viability/Cytotoxicity Assay Kit for Animal Live and Dead Cells (Biotium Inc., Fremont, CA, USA) was used to measure spheroid cell viability as per the manufacturer’s instructions. Calcein AM stains live cells, and ethidium homodimer III stains dead cells and yields green and red fluorescence, respectively. Fluorescent spheroid images were captured using the EVOS FL imaging system (Thermo Fisher Scientific, Waltham, MA, USA), and fluorescent quantification was performed using a SpectraMax iD3 microplate reader (Molecular Device) at 517 nm to detect Calcein AM and at 625 nm to detect EthD-III dye. Furthermore, the CellTiter-Glo 3D Cell Viability Assay (G968; Promega) dye was used according to the manufacturer’s instructions to determine the viability of spheroid cells. 

### 2.6. RNA Extraction and Quantitative Real-Time RT-PCR

Gene expression analysis was performed using the RT-qPCR method, as described previously [23]. NB cells were treated with different drug concentrations for 6 h. Total RNA was extracted by using RNeasy plus mini kit (74134; Qiagen, Hilden, Germany), followed by cDNA synthesis using a high-capacity cDNA reverse transcription kit (4368814; Thermo Fisher Scientific), as per manufacturer’s instructions. Further, the cDNA was used in RT-qPCR reactions for individual genes using SYBR Green dye (4385610; Thermo Fisher Scientific) and QuantStudio 3 Real-Time PCR System (Thermo Fisher Scientific). The expression of genes was normalized to the expression of GAPDH as a housekeeping gene. Primers used in this study are listed in Supplementary Table S1.

### 2.7. Immunoblotting Assays

Immunoblotting assays were performed by treating NB cells with different concentrations of CUDC-907 for 6 h, followed by cell lysis using RIPA buffer (89900; Thermo Fisher Scientific) supplemented with protease inhibitor cocktail (Complete mini EDTA free, Roche, Basel, Switzerland) and phosphatase inhibitor cocktail (PhosSTOP, Roche). Protein samples were quantified with Bradford assay (5000205; Bio-Rad, Hercules, CA, USA) and equal amounts of total protein were separated on 4–12% SDS-PAGE gels, followed by transfer to PVDF membranes using a Bio-Rad Trans-Blot Turbo TM system, then blocking with 5% BSA solution, and probing with corresponding primary antibodies (1:1000 dilution) overnight at 4 °C [25]. This was followed by washing and incubating the membranes with either anti-mouse or anti-rabbit IgG HRP-conjugated secondary antibody (1:10,000 dilution) for 2 h. Protein bands on membranes were developed using Clarity ECL Western substrate (Bio-Rad) and visualized using the ChemiDoc XRS Plus system (Bio-Rad). Densitometric analysis of the protein bands was performed using the ImageJ software.

### 2.8. Statistical Analysis

In the present study, three technical replicates and appropriate controls were performed for all the assays. Results are presented as the mean ± standard error mean (SEM) of the replicates. Two-tailed Student’s t-test and ANOVA statistical significance tests were used among different groups in experiments. The p values were calculated for fold differences among different comparative groups and *p* < 0.05 was considered statistically significant. Patient survival analyses were performed using the Kaplan–Meier method and two-sided log-rank tests.

## 3. Results

### 3.1. PI3K Expression Strongly Correlates with Poor NB Prognosis

**Figure 1:**
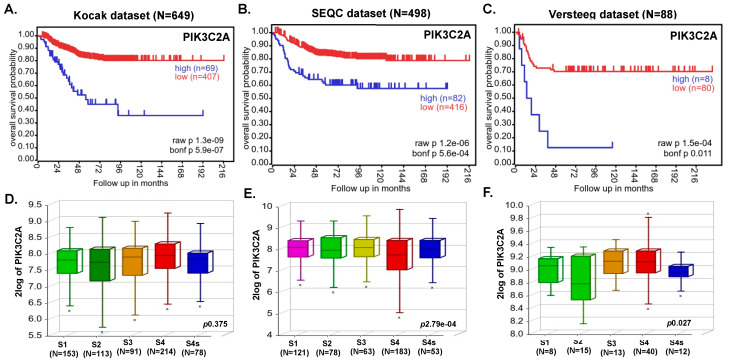
Expression of *PIK3C2A* gene inversely correlates with overall NB patients’ survival. Kaplan–Meier survival analysis of *PIK3C2A* gene expression shows an overall inverse probability of NB patients’ survival. (**A**) Kocak (N = 649). (**B**) SEQC (N = 498). (**C**) Versteeg (N = 88). (**D**–**F**) International Neuroblastoma Staging System (INSS)-based NB stage analysis showing the correlation of *PIK3C2A* expression with NB progression: (**D**) Kocak dataset, (**E**) SEQC dataset, (**F**) Versteeg dataset. x represents statistical significance within the group.

To investigate the role of PI3K in NB, we analyzed different NB patient datasets (total 1235 primary NB patients) using the R2 dataset and determined the correlation of the PI3K gene (*PIK3C2A*) with overall NB patient outcome. Kaplan–Meier survival analysis revealed that expression of the *PIK3C2A* gene inversely correlates with the overall survival of NB patients (Figure 1A–C). In contrast, the low expression of *PIK3C2A* gene showed significantly better prognosis and overall survival in all the analyzed patient datasets (Kocak N = 649, *p* = 1.3 × 10^−9^; SEQC N = 498, *p* = 1.2 × 10^−6^; Versteeg N = 88, *p* = 1.5 × 10^−4^; Figure 1A–C). Additionally, aggressive higher stage NB tumors showed higher *PIK3C2A* expression levels (Figure 1D–F), suggesting a role of PI3K in NB progression. Further, we observed a strong correlation between *PI3K* and *MYCN* genes in NB (data not shown). Additionally, *HDAC2* gene expression also showed an inverse correlation with overall and event-free survival of NB patients (Supplementary Figure S1). These findings suggest the oncogenic role of PI3K and HDAC in NB and highlight an effective therapeutic targeting strategy of using a dual inhibitor CUDC-907 for NB.

### 3.2. CUDC-907 Inhibits NB Proliferation

**Figure 2:**
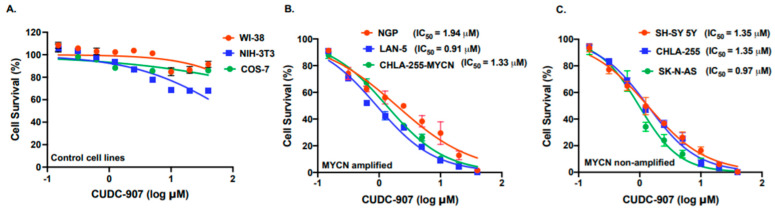
CUDC-907 inhibits NB proliferation. In vitro cytotoxicity assays in response to CUDC-907 using fibroblast cell lines and human NB cell lines. (**A**) WI-38, NIH-3T3, and COS-7 fibroblast cell lines. (**B**) NGP, LAN-5, and CHLA-255-MYCN as *MYCN*-amplified NB cell lines. (**C**) SH-SY-5Y, SK-N-AS, and CHLA-255 as *MYCN* non-amplified NB cell lines. The nonlinear variable slope regression method was used to determine IC_50_ values for individual NB cell lines.

**Figure 3:**
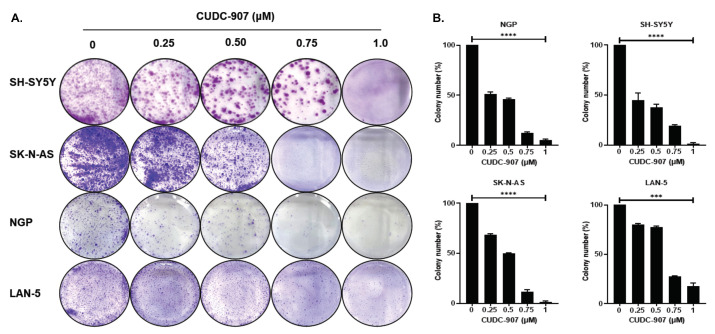
CUDC-907 inhibits NB colony formation and growth. Colony formation assays in response to CUDC-907 in four NB cell lines. (**A**) Representative images of colony formation assays in *MYCN*-amplified cell lines NGP and LAN-5, and *MYCN* non-amplified cell lines SH-SY-5Y and SK-N-AS in response to CUDC-907 treatment. Cells were treated with CUDC-907 for 48 h. (**B**) Survival index graphs showing the quantitation of the relative inhibition of colony formation in the respective cell lines in response to CUDC-907 treatment. Colony numbers are normalized to control. *** *p* < 0.001, **** *p* < 0.0001.

Based on the patient dataset analysis, we used CUDC-907, a dual PI3K and HDAC inhibitor in NB. Cytotoxicity assays for CUDC-907 in different NB cell lines and control fibroblast cell lines were performed (Figure 2). We used a total of six NB cell lines, including three *MYCN*-amplified and three *MYCN* non-amplified cell lines. Additionally, to determine the selectivity of CUDC-907 for cancer cells, we used three control fibroblast cell lines (Figure 2A). The results show that CUDC-907 significantly inhibits the cell proliferation of both *MYCN*-amplified (Figure 2B) and non-amplified cell lines (Figure 2C) in a dose-dependent manner and in contract to control fibroblast cell lines, with IC_50_ values ranging from 0.91 µM (*p* < 0.01) for LAN-5 to 1.94 (*p* < 0.05) µM for NGP (Figure 2). The results of the cytotoxicity assays on control cell lines clearly show the selectivity and potency of CUDC-907 in inhibiting NB proliferation (Figure 2A). To further validate the anti-proliferative effect of CUDC-907, we performed clonogenic assays using four different NB cell lines, including two *MYCN*-amplified (LAN-5, NGP) and two non-amplified (SH-SY5Y, SK-N-AS) cell lines. Our data showed that CUDC-907 significantly inhibits overall NB colony formation capacity in contrast to control treatment in a dose-dependent manner (Figure 3). These results further demonstrate the inhibitory effect of CUDC-907 on NB growth and proliferation.

### 3.3. CUDC-907 Induces Apoptosis and Blocks Cell Cycle Progression in NB

**Figure 4:**
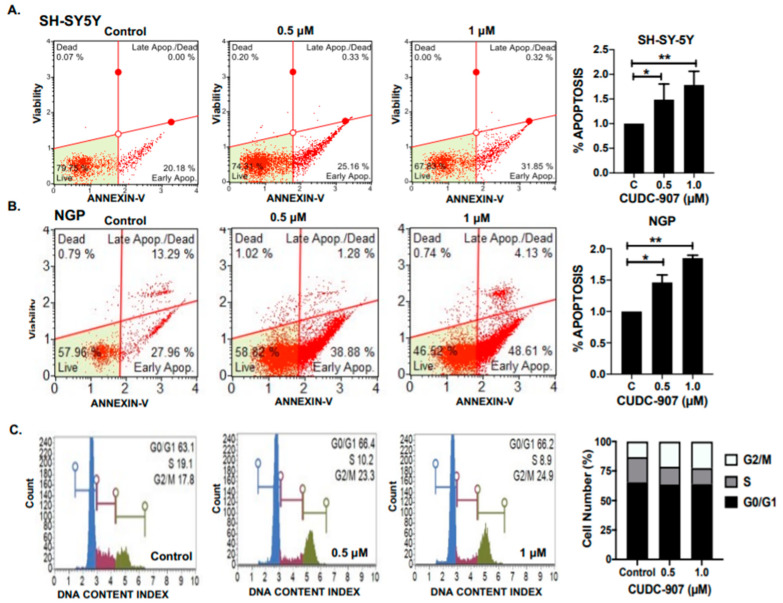
CUDC-907 induces apoptosis and blocks cell cycle progression in NB cells. (**A**,**B**) Representative images showing the percentage of apoptosis in NB cell lines in response to CUDC-907 treatment for 16 h. (**A**) SH-SY5Y and (**B**) NGP. Early apoptosis cells from three independent experiments are plotted and normalized to control treatments. C= control, * *p* < 0.05, ** *p* < 0.01. (**C**) Representative images of cell cycle assay in SH-SY5Y cell line in response to CUDC-907 treatment for 16 h. Percentage of cells in G0/G1, S, and G2/M cell cycle phases are represented, showing the effect of CUDC-907 on NB cell cycle phases.

Next, we performed apoptosis and cell cycle assays using different NB cell lines in response to CUDC-907. The results show that CUDC-907 significantly and in a dose-dependent manner induces apoptosis in both NGP and SH-SY5Y NB cell lines (Figure 4A,B). Specifically, CUDC-907 treatment increases the percentage of early apoptotic cells by 1.45- and 1.74-fold in SH-SY5Y cells, and 1.46- and 1.86-fold in NGP cells, in response to 0.5 µM and 1 µM, respectively, and in contrast to control treatment (Figure 4A,B).

Furthermore, we observed that CUDC-907 treatment significantly inhibits NB cell cycle progression by inhibiting the S phase (Figure 4C). In response to CUDC-907 treatment, the percentage of cells in the S phase decreased by 0.88- and 0.62-fold (*p* < 0.05), while the percentage of cells in the G2/M phase increased by 1.6- and 1.7-fold (*p* < 0.05), for 0.5 µM and 1 µM, respectively, and in contrast to control treatment (Figure 4C). These data further validate the efficacy of CUDC-907 in inducing cytotoxicity and inhibiting NB cell growth by arresting the cell cycle and by inducing apoptosis.

### 3.4. CUDC-907 Inhibits NB Spheroid Tumor Growth

**Figure 5:**
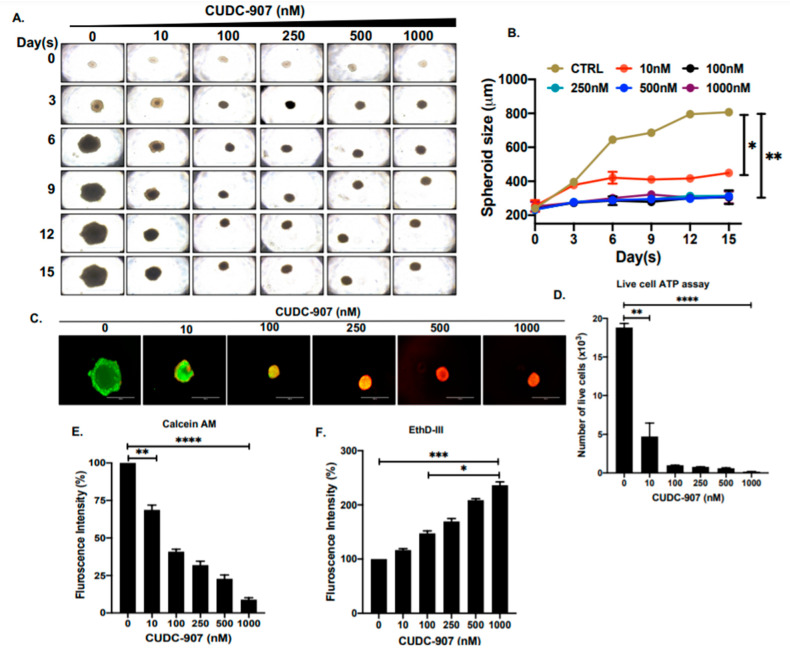
CUDC-907 inhibits NB spheroid tumor growth. (**A**) 3D spheroid tumors developed using SH-SY5Y cell line at different days of growth in response to increasing concentrations of CUDC-907 treatment. (**B**) Spheroid tumor growth measurements as displayed in (**A**), show a significant decrease in spheroidal mass in response to CUDC-907 treatments. (**C**) Representative images of terminal spheroids stained with Calcein AM (green; live cells) and EthD-III (red; dead cells) fluorescence dyes. (**D**) Quantitative assessment of the number of live cells in 3D spheroid tumors using live-cell ATP release assay. (**E**) Quantitative representation of the percentage of cells stained with Calcein AM shows a significant decrease in the number of live cells. (**F**) Quantitative representation of the percentage of cells stained with EthD-III shows a significant increase in the number of dead cells. * *p* < 0.05, ** *p* < 0.01. *** *p* < 0.001, **** *p* < 0.0001.

To further determine the effect of CUDC-907, we developed a 3D spheroid tumor model of NB by using the SH-SY5Y cell line. These 3D spheroids strongly mimic the in vivo physiological growth patterns of solid tumor NB by generating anchorage-independent spheroid tumor mass. We have developed spheroid tumors of similar sizes, randomized them, and treated them with increasing doses of CUDC-907 (Figure 5). The size and growth of individual spheroid tumors were measured and imaged regularly. The results show significant and dose-dependent inhibition of NB tumor growth in response to CUDC-907 and in contrast to control treatment (Figure 5A,B). Furthermore, we observed a significant reduction of live cells in spheroid tumors in response to CUDC-907 treatments, emphasizing the effect of CUDC-907 in inhibiting tumor mass by blocking NB cell growth (Figure 5C,E,F). These results were further validated by a live-cell ATP release assay, which demonstrated that CUDC-907 significantly and in a dose-dependent manner inhibits the number of live cells in NB spheroid tumors to overall inhibiting tumor growth (Figure 5D). These qualitative and quantitative assays in 3D spheroid tumors further confirmed the efficacy of CUDC-907 as a single drug in inhibiting NB growth.

### 3.5. CUDC-907 Inhibits PI3K/AKT Pathway

**Figure 6:**
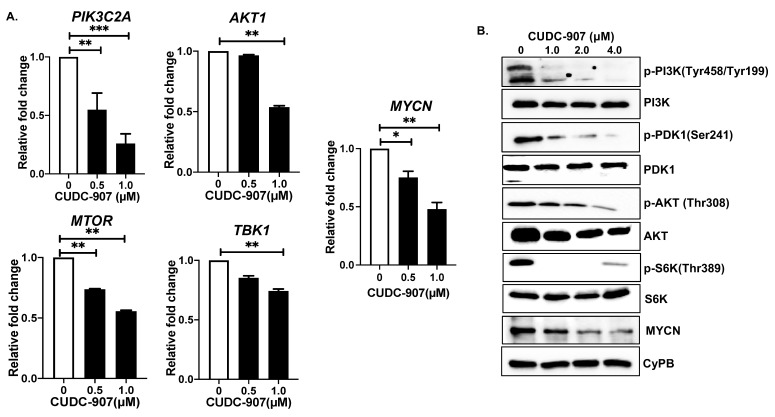
CUDC-907 inhibits PI3K/AKT pathway: (**A**) Gene expression analysis of *PIK3C2A*, *AKT1*, *MTOR*, *TBK1*, and *MYCN* in response to CUDC-907 treatments in SH-SY5Y cells. * *p* < 0.05, ** *p* < 0.01, and *** *p* < 0.001. (**B**) Western blot analysis of different PI3K pathway proteins in response to increasing concentrations of CUDC-907 treatments. CyPB was used as a loading control. Full blots and densitometric data are available in Supplementary Figure S2.

To further determine the molecular mechanisms by which CUDC-907 inhibits NB growth, we performed gene and protein expression profiling. Gene expression analysis showed that CUDC-907 significantly inhibits mRNA expression of key PI3K/AKT pathway genes, including *PIK3C2A*, *AKT1*, *TBK1*, and *MTOR* (Figure 6A). For the *PIK3C2A* gene, we observed about three-fold inhibition of mRNA expression in response to 1 µM of CUDC-907, and in contrast to the control treatment (Figure 6A). Additionally, we found that CUDC-907 leads to significant inhibition of the phosphorylation and activation of the PI3K/AKT pathway proteins, including p-PI3K (Tyr458/Tyr199), p-PDK1 (S241), p-AKT (T308), and p-S6K (T389). The dose-dependent inhibition of phosphorylation was observed in response to CUDC-907 treatment and in contrast to the control treatment (Figure 6B). As expected, the total protein levels of PI3K, PDK1, AKT, and S6K were found to be slightly reduced in response to CUDC-907 treatments (Figure 6B). Furthermore, we observed a dose-dependent inhibition of MYCN expression at both mRNA and protein levels in response to the CUDC-907 treatment (Figure 6A,B). These data highlight the effects of CUDC-907 in inhibiting the PI3K/AKT pathway and MYCN at both mRNA and protein levels.

### 3.6. CUDC-907 Inhibits HDAC and Induces Histone Acetylation at H3K9

**Figure 7:**
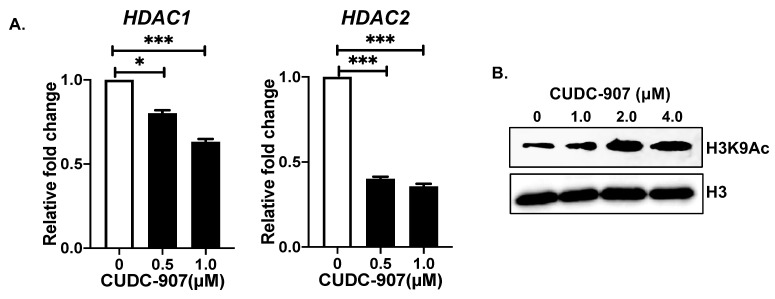
CUDC-907 inhibits HDAC: (**A**) Gene expression analysis of *HDAC1* and *HDAC2* in response to CUDC-907 treatments in SH-SY5Y cells. * *p* < 0.05, *** *p* < 0.001. (**B**) Western blot analysis of H3K9Ac and total H3 in response to increasing concentrations of CUDC-907 treatments. H3 was used as a loading control. Full blots and densitometric data are available in Supplementary Figure S2.

To further understand the effect of CUDC-907 at the epigenetic level, we analyzed the gene expression of histone deacetylases *HDAC1* and *HDAC2* in response to CUDC-907. Our results show an inhibition of *HDAC1* mRNA by 0.2- and 0.4-fold and *HDAC2* mRNA by 0.6- and 0.7-fold in response to 0.5 and 1.0 µM, respectively, and in contrast to control treatment (Figure 7A). Additionally, to determine the effect of HDAC inhibition, we further analyzed the overall H3K9Ac levels in NB cells. The results show that CUDC-907 in a dose-dependent manner significantly increases H3K9Ac levels in contrast to the control (Figure 7B). These data highlight the epigenetic effects of CUDC-907 by inhibiting HDACs and therefore enhancing H3K9Ac levels in NB.

## 4. Discussion

The direct targeting of PI3K and HDAC enzymes has been shown as an effective therapeutic approach in various cancers, including NB [7,26,27]. To date, five PI3K inhibitors (Copanlisib, Idelalisib, Umbralisib, Duvelisib, and Alpelisib) and four HDAC inhibitors (Romidepsin, Vorinostat, Belinostat, and Panobinostat) received FDA approval for treating different types of leukemia and lymphoma, multiple myeloma, and breast cancer [28,29]. This highlights the clinical importance of both PI3K and HDAC inhibitors for the development of targeted therapeutic approaches for hard-to-treat cancers, such as NB [28]. A combination of PI3K and HDAC inhibitors has been shown to synergistically inhibit tumor cell growth and induce apoptosis in diffuse large B-cell lymphoma and glioblastoma multiforme [30]. Drug combinational approaches require individual administration of two or more drugs for their targets and are expected to have synergistic or additive effects [31]. Another combination therapeutic approach is to develop single-agent dual inhibitor drugs that can target two different pathways simultaneously. This approach has several advantages, such as low toxicity, greater therapeutic efficacy, better pharmacokinetic and pharmacodynamic properties, and fewer solubility-related and drug–drug interaction related issues [18].

In the present study, we used a dual inhibitor CUDC-907 that targets both PI3K and HDAC [19,32]. Pre-clinical studies in colorectal cancer, B cell lymphoma, thyroid cancer, refractory lymphoma, and multiple myeloma have shown that CUDC-907 significantly inhibits both the PI3K pathway and different HDAC enzymes, to inhibit cancer proliferation and growth [33,34]. Similarly, our results in NB also showed that CUDC-907, in a dose-dependent manner, inhibits NB cell proliferation and 3D spheroid tumor growth. CUDC-907 has been reported to inhibit pancreatic adenocarcinoma [35], glioblastoma [36], prostate cancer [21], and acute myeloid leukemia [20] by inhibiting HDACs, DNA damage response, cell cycle proteins, and by inducing apoptosis. In NB, we demonstrated that CUDC-907 induces apoptosis and inhibits the cell cycle S phase. Similar results of the CUDC-907-mediated blockage of the S and G2/M phases were shown for pancreatic and thyroid cancers via the downregulation of the cell cycle regulators cyclin B1, AURKA, and PLK1 [33,35]. In glioblastoma, CUDC-907 induces G1 cell cycle arrest through *CDKN1A* promoter hyperacetylation-driven transcriptional activation and downregulation of CDK1 [36], while in lung fibroblast cells, CUDC-907 blocks G1 and S phase [37]. In breast cancer, CUDC-907 enhances TRAIL-induced apoptosis through the upregulation of cell survival proteins, including XIAP, Bcl-xL, and Bcl-2 [38]. The oncogenic activation of PI3K/AKT signaling pathway regulates multiple parallel signaling pathways, which are known to be involved in metabolism, proliferation, motility, and autophagy in different cancers [39,40]. Our results also showed that CUDC-907-mediated PI3K and HDAC inhibition leads to inhibiting NB growth.

In the past decade, most preclinical studies were based on the 2D cell culture models despite its limitations, including difficulty in simulating three-dimensional physiological conditions [41,42]. In vitro 3D tumor models filled this gap by enumerating the growth patterns of in vivo solid tumors. These 3D spheroidal tumor models became a research standard by replacing time-consuming and expensive animal studies to determine preliminary anti-cancer drug effects [42,43]. Three-dimensional cell culture models have higher invasiveness and resistance to cytotoxic drugs in comparison to two-dimensional monolayer studies [44]. Three-dimensional spheroidal tumor studies were shown as a substitute for in vivo animal models in different cancers, including NB [45]. In our study, we observed a dose-dependent inhibition of NB spheroidal tumor growth by CUDC-907. Similar results were observed in in vitro hepatocarcinoma, pancreatic cancer, and thyroid cancer spheroidal models, and were found comparable with in vivo studies in these cancers [17,33,35]. Our results demonstrated the effect of CUDC-907 in inhibiting the PI3K/AKT, HDACs, and enhancing the H3K9Ac levels in NB. Numerous reports in different cancers have shown similar results of the CUDC-907-mediated inhibition of PI3K, HDACs, and related proteins [20,21,32,35,37,38]. *MYCN* is one of the most important prognostic factors for the NB progression [46]. Our patient dataset analysis revealed a strong correlation of *PI3K* and *MYCN* levels in primary NB patients. *PI3K* expression inversely correlates with the poor overall survival of NB patients, and *PI3K* expression is found to be significantly high in *MYCN* amplified tumors. Similarly, *HDAC2* expression has been shown to correlate with MYCN levels, and multiple reports show the development of alternative strategies to target MYCN via the HDAC inhibition [6]. These analyses indicate that the dual targeting of PI3K and HDAC by CUDC-907 may inhibit MYCN levels. Our results showed that CUDC-907 significantly inhibits MYCN at both gene and protein levels. Similar results have been shown for the CUDC-907-mediated inhibition of cancer cell growth by inhibiting MYC levels in pancreatic adenocarcinoma, acute myeloid leukemia, and prostate cancer [20,21,35].

Overall, our results showed the utility of drug repurposing by demonstrating the efficacy of CUDC-907 in inhibiting NB growth. CUDC-907 is currently in phase I and phase II clinical trials for different tumor types, including pediatric cancers (NCT02307240, NCT02674750, NCT02909777, and NCT03002623). In phase I clinical studies, CUDC-907 has been considered as a moderately safe compound with minor gastrointestinal and hematologic side effects [47,48]. Therefore, our current study will provide further preclinical evidence for repurposing CUDC-907 for NB, to develop a novel and effective therapeutic approach for NB patients.

## 5. Conclusions

Our present study highlights the efficacy and potency of the dual inhibitor CUDC-907 in inhibiting NB growth. Our data demonstrated the inverse correlation between PI3K and HDAC levels and overall NB patient outcomes. To conclude, CUDC-907 as a single drug directly targets both PI3K and HDAC to inhibit the PI3K/AKT cell signaling pathway and to enhance H3K9Ac levels that lead to the downregulation of MYCN and inhibition of overall NB growth. As CUDC-907 is currently under advanced clinical trials for both pediatric and adult cancers, our pre-clinical results will further pave the way for a successful clinical translation of CUDC-907 for treating NB patients.

## Data Availability

Publicly available datasets were analyzed in this study. This data can be found at R2 Genomic Analysis and Visualization Platform. Available online: https://hgserver1.amc.nl/cgi-bin/r2/main.cgi (accessed on 1 February 2022).

## Supplementary Materials

The following supporting information can be downloaded at: https://www.mdpi.com/article/10.3390/cancers14041067/s1: Figure S1: *HDAC2* expression correlates with poor overall and event-free survival of NB patients; Figure S2: Full blots and densitometric analysis of Western blots; Table S1: RT-qPCR primers used in this study.

## References

[1] Louis C.U., Shohet J.M. (2015). Neuroblastoma: Molecular pathogenesis and therapy. Annu. Rev. Med..

[2] Smith V., Foster J. (2018). High-Risk Neuroblastoma Treatment Review. Children.

[3] Zafar A., Wang W., Liu G., Wang X., Xian W., McKeon F., Foster J., Zhou J., Zhang R. (2021). Molecular targeting therapies for neuroblastoma: Progress and challenges. Med. Res. Rev..

[4] Puissant A., Frumm S.M., Alexe G., Bassil C.F., Qi J., Chanthery Y.H., Nekritz E.A., Zeid R., Gustafson W.C., Greninger P. (2013). Targeting MYCN in neuroblastoma by BET bromodomain inhibition. Cancer Discov..

[5] Canete A., Gerrard M., Rubie H., Castel V., Di Cataldo A., Munzer C., Ladenstein R., Brichard B., Bermudez J.D., Couturier J. (2009). Poor survival for infants with MYCN-amplified metastatic neuroblastoma despite intensified treatment: The International Society of Paediatric Oncology European Neuroblastoma Experience. J. Clin. Oncol..

[6] Phimmachanh M., Han J.Z.R., O’Donnell Y.E.I., Latham S.L., Croucher D.R. (2020). Histone Deacetylases and Histone Deacetylase Inhibitors in Neuroblastoma. Front. Cell Dev. Biol..

[7] Yang J., Nie J., Ma X., Wei Y., Peng Y., Wei X. (2019). Targeting PI3K in cancer: Mechanisms and advances in clinical trials. Mol. Cancer.

[8] Papa A., Pandolfi P.P. (2019). The PTEN(-)PI3K Axis in Cancer. Biomolecules.

[9] Nalairndran G., Hassan Abdul Razack A., Mai C.W., Fei-Lei Chung F., Chan K.K., Hii L.W., Lim W.M., Chung I., Leong C.O. (2020). Phosphoinositide-dependent Kinase-1 (PDPK1) regulates serum/glucocorticoid-regulated Kinase 3 (SGK3) for prostate cancer cell survival. J. Cell. Mol. Med..

[10] Balasuriya N., Davey N.E., Johnson J.L., Liu H., Biggar K.K., Cantley L.C., Li S.S., O’Donoghue P. (2020). Phosphorylation-dependent substrate selectivity of protein kinase B (AKT1). J. Biol. Chem..

[11] Shorning B.Y., Dass M.S., Smalley M.J., Pearson H.B. (2020). The PI3K-AKT-mTOR Pathway and Prostate Cancer: At the Crossroads of AR, MAPK, and WNT Signaling. Int. J. Mol. Sci..

[12] Ghoneum A., Said N. (2019). PI3K-AKT-mTOR and NFkappaB Pathways in Ovarian Cancer: Implications for Targeted Therapeutics. Cancers.

[13] Wang P., Wang Z., Liu J. (2020). Role of HDACs in normal and malignant hematopoiesis. Mol. Cancer.

[14] Li Y., Seto E. (2016). HDACs and HDAC Inhibitors in Cancer Development and Therapy. Cold Spring Harb. Perspect. Med..

[15] Lemoine M., Younes A. (2010). Histone deacetylase inhibitors in the treatment of lymphoma. Discov. Med..

[16] Rahmani M., Yu C., Reese E., Ahmed W., Hirsch K., Dent P., Grant S. (2003). Inhibition of PI-3 kinase sensitizes human leukemic cells to histone deacetylase inhibitor-mediated apoptosis through p44/42 MAP kinase inactivation and abrogation of p21(CIP1/WAF1) induction rather than AKT inhibition. Oncogene.

[17] Liao W., Yang W., Xu J., Yan Z., Pan M., Xu X., Zhou S., Zhu Y., Lan J., Zeng M. (2021). Therapeutic Potential of CUDC-907 (Fimepinostat) for Hepatocarcinoma Treatment Revealed by Tumor Spheroids-Based Drug Screening. Front. Pharmacol..

[18] Ranganna K., Selvam C., Shivachar A., Yousefipour Z. (2020). Histone Deacetylase Inhibitors as Multitarget-Directed Epi-Drugs in Blocking PI3K Oncogenic Signaling: A Polypharmacology Approach. Int. J. Mol. Sci..

[19] Qiao X., Ma J., Knight T., Su Y., Edwards H., Polin L., Li J., Kushner J., Dzinic S.H., White K. (2021). The combination of CUDC-907 and gilteritinib shows promising in vitro and in vivo antileukemic activity against FLT3-ITD AML. Blood Cancer J..

[20] Li X., Su Y., Madlambayan G., Edwards H., Polin L., Kushner J., Dzinic S.H., White K., Ma J., Knight T. (2019). Antileukemic activity and mechanism of action of the novel PI3K and histone deacetylase dual inhibitor CUDC-907 in acute myeloid leukemia. Haematologica.

[21] Hu C., Xia H., Bai S., Zhao J., Edwards H., Li X., Yang Y., Lyu J., Wang G., Zhan Y. (2020). CUDC-907, a novel dual PI3K and HDAC inhibitor, in prostate cancer: Antitumour activity and molecular mechanism of action. J. Cell Mol. Med..

[22] Agarwal S., Lakoma A., Chen Z., Hicks J., Metelitsa L.S., Kim E.S., Shohet J.M. (2015). G-CSF Promotes Neuroblastoma Tumorigenicity and Metastasis via STAT3-Dependent Cancer Stem Cell Activation. Cancer Res..

[23] Chilamakuri R., Rouse D.C., Yu Y., Kabir A.S., Muth A., Yang J., Lipton J.M., Agarwal S. (2022). BX-795 inhibits neuroblastoma growth and enhances sensitivity towards chemotherapy. Transl. Oncol..

[24] Guan S., Lu J., Zhao Y., Yu Y., Li H., Chen Z., Shi Z., Liang H., Wang M., Guo K. (2018). MELK is a novel therapeutic target in high-risk neuroblastoma. Oncotarget.

[25] Agarwal S., Ghosh R., Chen Z., Lakoma A., Gunaratne P.H., Kim E.S., Shohet J.M. (2016). Transmembrane adaptor protein PAG1 is a novel tumor suppressor in neuroblastoma. Oncotarget.

[26] Stopsack K.H., Huang Y., Tyekucheva S., Gerke T.A., Bango C., Elfandy H., Bowden M., Penney K.L., Roberts T.M., Parmigiani G. (2020). Multiplex Immunofluorescence in Formalin-Fixed Paraffin-Embedded Tumor Tissue to Identify Single-Cell-Level PI3K Pathway Activation. Clin. Cancer Res..

[27] Mohlin S., Hansson K., Radke K., Martinez S., Blanco-Apiricio C., Garcia-Ruiz C., Welinder C., Esfandyari J., O’Neill M., Pastor J. (2019). Anti-tumor effects of PIM/PI3K/mTOR triple kinase inhibitor IBL-302 in neuroblastoma. EMBO Mol. Med..

[28] Brown J.R. (2019). Phosphatidylinositol 3 Kinase delta Inhibitors: Present and Future. Cancer J..

[29] Raedler L.A. (2016). Farydak (Panobinostat): First HDAC Inhibitor Approved for Patients with Relapsed Multiple Myeloma. Am. Health Drug Benefits.

[30] Meng W., Wang B., Mao W., Wang J., Zhao Y., Li Q., Zhang C., Ma J. (2019). Enhanced efficacy of histone deacetylase inhibitor panobinostat combined with dual PI3K/mTOR inhibitor BEZ235 against glioblastoma. Nagoya J. Med. Sci..

[31] De Lera A.R., Ganesan A. (2016). Epigenetic polypharmacology: From combination therapy to multitargeted drugs. Clin. Epigenetics.

[32] Ma L., Bian X., Lin W. (2021). Correction to: The dual HDAC-PI3K inhibitor CUDC-907 displays single-agent activity and synergizes with PARP inhibitor olaparib in small cell lung cancer. J. Exp. Clin. Cancer Res..

[33] Kotian S., Zhang L., Boufraqech M., Gaskins K., Gara S.K., Quezado M., Nilubol N., Kebebew E. (2017). Dual Inhibition of HDAC and Tyrosine Kinase Signaling Pathways with CUDC-907 Inhibits Thyroid Cancer Growth and Metastases. Clin. Cancer Res..

[34] Zhong L., Li Y., Xiong L., Wang W., Wu M., Yuan T., Yang W., Tian C., Miao Z., Wang T. (2021). Small molecules in targeted cancer therapy: Advances, challenges, and future perspectives. Signal. Transduct. Target. Ther..

[35] Fu X.H., Zhang X., Yang H., Xu X.W., Hu Z.L., Yan J., Zheng X.L., Wei R.R., Zhang Z.Q., Tang S.R. (2019). CUDC-907 displays potent antitumor activity against human pancreatic adenocarcinoma in vitro and in vivo through inhibition of HDAC6 to downregulate c-Myc expression. Acta Pharmacol. Sin..

[36] Pal S., Kozono D., Yang X., Fendler W., Fitts W., Ni J., Alberta J.A., Zhao J., Liu K.X., Bian J. (2018). Dual HDAC and PI3K Inhibition Abrogates NFkappaB- and FOXM1-Mediated DNA Damage Response to Radiosensitize Pediatric High-Grade Gliomas. Cancer Res..

[37] Zhang W., Zhang Y., Tu T., Schmull S., Han Y., Wang W., Li H. (2020). Dual inhibition of HDAC and tyrosine kinase signaling pathways with CUDC-907 attenuates TGFbeta1 induced lung and tumor fibrosis. Cell Death Dis..

[38] Li Z.J., Hou Y.J., Hao G.P., Pan X.X., Fei H.R., Wang F.Z. (2020). CUDC-907 enhances TRAIL-induced apoptosis through upregulation of DR5 in breast cancer cells. J. Cell Commun. Signal..

[39] Westhoff M.A., Faham N., Marx D., Nonnenmacher L., Jennewein C., Enzenmuller S., Gonzalez P., Fulda S., Debatin K.M. (2013). Sequential dosing in chemosensitization: Targeting the PI3K/Akt/mTOR pathway in neuroblastoma. PLoS ONE.

[40] Lin A., Piao H.L., Zhuang L., Sarbassov D.D., Ma L., Gan B. (2014). FoxO transcription factors promote AKT Ser473 phosphorylation and renal tumor growth in response to pharmacologic inhibition of the PI3K-AKT pathway. Cancer Res..

[41] Jensen C., Teng Y. (2020). Is It Time to Start Transitioning From 2D to 3D Cell Culture?. Front. Mol. Biosci..

[42] Daunys S., Janoniene A., Januskeviciene I., Paskeviciute M., Petrikaite V. (2021). 3D Tumor Spheroid Models for In Vitro Therapeutic Screening of Nanoparticles. Adv. Exp. Med. Biol..

[43] Gilazieva Z., Ponomarev A., Rutland C., Rizvanov A., Solovyeva V. (2020). Promising Applications of Tumor Spheroids and Organoids for Personalized Medicine. Cancers.

[44] Edmondson R., Broglie J.J., Adcock A.F., Yang L. (2014). Three-dimensional cell culture systems and their applications in drug discovery and cell-based biosensors. Assay Drug Dev. Technol..

[45] Kumar A., Fan D., Dipette D.J., Singh U.S. (2014). Sparstolonin B, a novel plant derived compound, arrests cell cycle and induces apoptosis in N-myc amplified and N-myc nonamplified neuroblastoma cells. PLoS ONE.

[46] Schneiderman J., London W.B., Brodeur G.M., Castleberry R.P., Look A.T., Cohn S.L. (2008). Clinical significance of MYCN amplification and ploidy in favorable-stage neuroblastoma: A report from the Children’s Oncology Group. J. Clin. Oncol..

[47] Oki Y., Kelly K.R., Flinn I., Patel M.R., Gharavi R., Ma A., Parker J., Hafeez A., Tuck D., Younes A. (2017). CUDC-907 in relapsed/refractory diffuse large B-cell lymphoma, including patients with MYC-alterations: Results from an expanded phase I trial. Haematologica.

[48] Chen I.C., Sethy B., Liou J.P. (2020). Recent Update of HDAC Inhibitors in Lymphoma. Front. Cell Dev. Biol..

