# Supplementary material for: Dual Targeting of PI3K and HDAC by CUDC-907 Inhibits Pediatric Neuroblastoma Growth
Source: Cancers (Basel). 2022 Feb 20;14(4):1067. doi: 10.3390/cancers14041067 (PMC8870466; doi:10.3390/cancers14041067)
Supplement: Supplementary file 1 [file cancers-14-01067-s001.zip › Supplementary.pdf]

Supplementary Figure S1

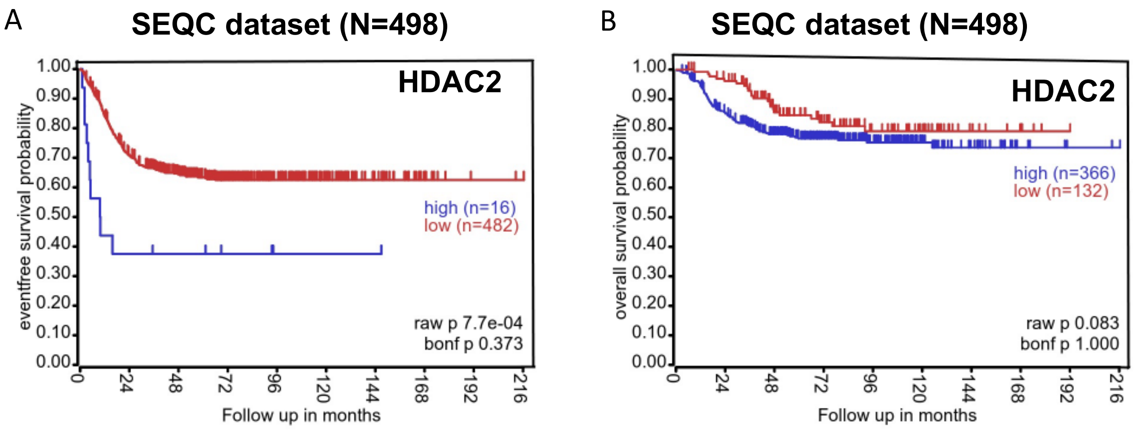

Supplementary Figure S1: *HDAC2* expression correlates with poor overall and event-free survival of NB patients. Kaplan-Meier analysis of *HDAC2* gene expression showing **(A)** event-free and **(B)** overall survival probability of NB patients' survival in SEQC dataset of 498 patients.

Supplementary Figure S2:

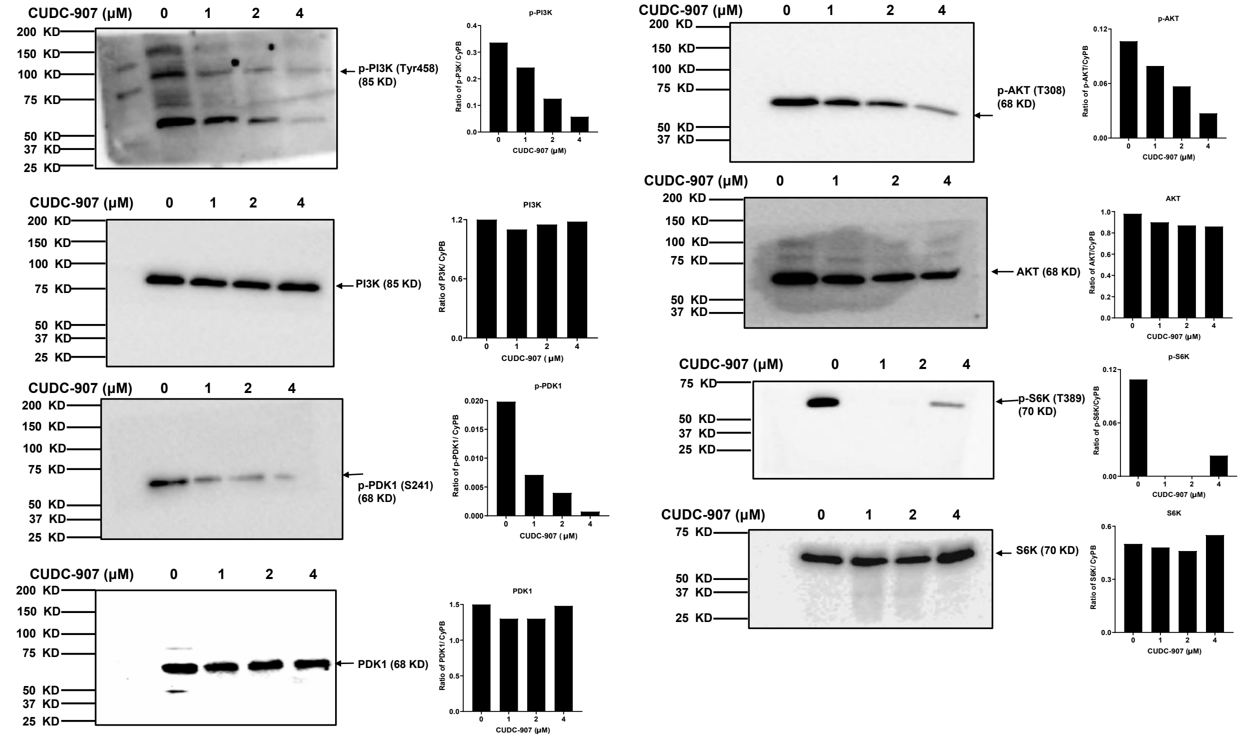

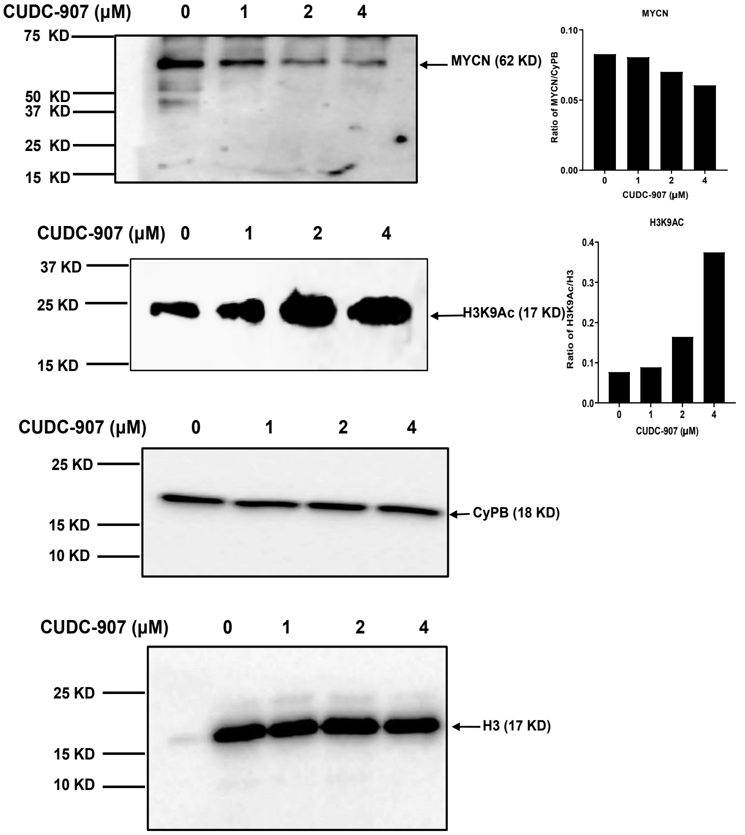

Supplementary Figure S2: Full blots of the immunoblotting results presented in this study as Figure 6B and Figure 7. Protein molecular weight marker, size of the expected band, and name of the protein are indicated with all blots. Protein band densitometric analysis was performed using the ImageJ software and is shown next to each blot for the indicated band.

Supplementary Table 1: RT-qPCR primers used in this study

Gene	**Forward primer (5'-3')**	**Reverse primer (5'-3')**
PIK3C2A	CTGCCTGCGACAGATGAGTG	TCCGATTACCAAGTGCTCTTTC
AKT1	GCACAAACGAGGGGAGTACA	AAGGTCCGTTCGATGACAGT
TBK1	CTTGCAGTCTTTCTCGGGGT	CTGGTCAAAACCCCAACACT
MTOR	TCTTGGCCATAGCTAGCCTC	CAACTAGGTCATTGGAGGGG
HDAC1	CGCCCTCACAAAGCCAATG	CTGCTTGCTGTACTCCGACA
HDAC2	ATGGCGTACAGTCAAGGAGG	TGCGGATTCTATGAGGCTTCA
MYCN	ACCCTGAGCGATTCAGATGA	CCACAGTGACCACGTCGATT
GAPDH	CACCATCTTCCAGGAGCGAG	TGATGACCCTTTTGGCTCCC
